# Nucleotide augmentation for machine learning-guided protein engineering

**DOI:** 10.1093/bioadv/vbac094

**Published:** 2022-12-09

**Authors:** Mason Minot, Sai T Reddy

**Affiliations:** Department of Biosystems Science and Engineering, ETH Zurich, 4058 Basel, Switzerland; Department of Biosystems Science and Engineering, ETH Zurich, 4058 Basel, Switzerland

## Abstract

**Summary:**

Machine learning-guided protein engineering is a rapidly advancing field. Despite major experimental and computational advances, collecting protein genotype (sequence) and phenotype (function) data remains time- and resource-intensive. As a result, the quality and quantity of training data are often a limiting factor in developing machine learning models. Data augmentation techniques have been successfully applied to the fields of computer vision and natural language processing; however, there is a lack of such augmentation techniques for biological sequence data. Towards this end, we develop nucleotide augmentation (NTA), which leverages natural nucleotide codon degeneracy to augment protein sequence data via synonymous codon substitution. As a proof of concept for protein engineering, we test several online and offline augmentation implementations to train machine learning models with benchmark datasets of protein genotype and phenotype, revealing performance gains on par and surpassing benchmark models using a fraction of the training data. NTA also enables substantial improvements for classification tasks under heavy class imbalance.

**Availability and implementation:**

The code used in this study is publicly available at https://github.com/minotm/NTA

**Supplementary information:**

Supplementary data are available at *Bioinformatics Advances* online.

## 1 Introduction

The application of machine learning (ML) on biological sequence data has expanded substantially in recent years (Angermueller *et al.*, 2016; Jurtz *et al.*, 2017). One area of interest is ML-guided protein engineering, which enables efficient and large-scale exploration of protein sequence space beyond what is possible by experimental screening alone (Yang *et al.*, 2019). This approach has been used for a variety of applications such as increasing protein expression (Shin *et al.*, 2021), multi-parameter optimization of antibody therapeutics (Mason *et al.*, 2021), improving the thermostability and function of enzymes (Biswas *et al.*, 2021; Romero *et al.*, 2013) and predicting SARS-CoV-2 escape variants (Taft *et al.*, 2022).

ML-guided protein engineering has been supported in recent years by advances in DNA synthesis, high-throughput screening assays and deep sequencing, which enable the generation of phentoype–genotype training data. However, collecting protein sequence and function (labeled) data still remains time- and resource-intensive. As is the case in other applications of ML, small or imbalanced training datasets can often lead to bias and overfitting, thereby leading to a lack of performance and generalizability (Shorten and Khoshgoftaar, 2019). Moreover, large-scale datasets are often required to train effective deep-learning models (Halevy *et al.*, 2009; Sun *et al.*, 2017). Several approaches have been developed to make the most of limited data. For example, Wittmann *et al.* (2021b) show that ML-informed design of training datasets can improve directed evolution workflows. Additionally, language models trained on large-scale and mostly natural sequence data have been developed to generate protein embeddings capable of improving downstream tasks like secondary structure and contact map prediction (Biswas *et al.*, 2021; Luo *et al.*, 2021; Ofer *et al.*, 2021; Rao *et al.*, 2021, 2019).

In the ML-related fields of computer vision (Perez and Wang, 2017; Shorten and Khoshgoftaar, 2019; Taylor and Nitschke, 2018) and natural language processing (NLP) (Anaby-Tavor *et al.*, 2020; Feng *et al.*, 2021; Sennrich *et al.*, 2016), data augmentation is applied to combat data limitations. Data augmentation refers to techniques that artificially increase the number of training examples, which can lead to improved performance and act as a regularizer in reducing overfitting. Common image augmentation approaches include copying and warping an image, i.e. via cropping and rotation (Shorten and Khoshgoftaar, 2019). NLP augmentation techniques may include copying a sentence and substituting words with synonyms to preserve meaning or translating a sentence into another language and back again (Anaby-Tavor *et al.*, 2020; Feng *et al.*, 2021; Sennrich *et al.*, 2016). Additionally, synthetic data can be generated through a variety of techniques including Generative Adversarial Networks (GANs) and the Synthetic Minority Oversampling Technique (SMOTE) (Chawla *et al.*, 2002; Shorten and Khoshgoftaar, 2019).

Recently, data augmentation techniques have also been developed for protein sequence data. Such approaches include GANs (Han *et al.*, 2019; Li and Zhang, 2022) and augmentation for protein language models with contrastive learning via evolutionary information and string manipulations such as amino acid replacement and sequence shuffling (Lu *et al.*, 2020; Shen *et al.*, 2021). While certainly noteworthy, these approaches are constrained to the domain in which they were trained (GANs) or may be less relevant in protein engineering workflows that generate large libraries of non-natural mutations in a small number of residues of a single protein (Biswas *et al.*, 2021; Dallago *et al.*, 2021; Wittmann *et al.*, 2021a) (language models). Unlike computer vision and NLP, there exists a lack of simple, easy-to-apply data augmentation techniques for protein sequence data; likely resulting from the relationship between the discrete amino acid sequence of a protein and its structure and function.

**Fig. 1.**
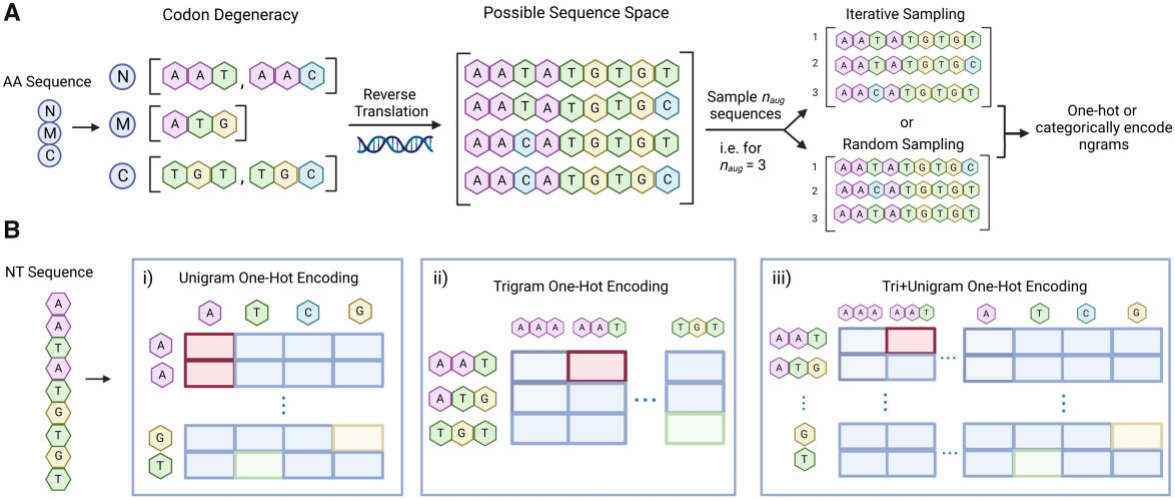
(**A**) Nucleotide augmentation (NTA) approach. Possible nucleotide codons are determined for each residue in an input amino acid sequence. Full-length nucleotide sequences are then sampled *n*_aug_ times from the possible sequence space via iterative or random sampling, where *n*_aug_ is a user-specified augmentation factor. The sequences are then either categorically encoded (Transformer) or one-hot encoded (CNN). The different ngram one-hot encoding approaches are illustrated in (**B**). First, a nucleotide sequence is broken into (B_i_) unigrams (B_ii_) trigrams or (B_iii_) trigrams concatenated with unigrams (tri+unigrams). The ngrams are then tokenized according to their respective vocabularies. The tokenized vector corresponds to the Transformer model’s input; however, this vector undergoes an additional one-hot encoding step for the CNN. For example, the resulting one-hot matrix size for a nucleotide sequence of length 9 will either be 9 × 4 (unigrams B_i_), 3 × 64 (trigrams, B_ii_) or 12 × 68 (tri+unigrams B_iii_). Note that amino acids are encoded as unigrams i.e. for sequence length L the resulting one-hot matrix is L×20 for the 20 canonical amino acids. Created with Biorender.com

Here, we establish nucleotide augmentation (NTA), which represents a rapid and facile data augmentation technique for ML-guided protein engineering. By taking advantage of natural codon degeneracy (trinucleotide combinations), we develop NTA as the reverse translation of an amino acid sequence into multiple, unique nucleotide sequences via codon degeneracy (Fig. 1). Additionally, we develop and characterize offline and online implementations of the concept (Algorithms 1 and 2). To benchmark and validate the performance of NTA, we select three protein engineering datasets with various sizes of training data and class balances (Bryant *et al.*, 2021; Dallago *et al.*, 2021; Mason *et al.*, 2021; Wu *et al.*, 2016). Using benchmarked train/test splits, we determine that NTA can improve predictive performance of ML models with limited training data and under high-class imbalance.

## 2 Methods

### 2.1 Datasets

In order to evaluate the performance of NTA for ML-guided protein engineering, we acquired three benchmark-labeled datasets. Such datasets consist of protein mutagenesis libraries where each sequence variant (genotype) in the library and its corresponding function (phenotype) are known. High-throughput screening by directed evolution coupled with deep sequencing provides an exemplary approach to generate such datasets. Although protein engineering has largely lacked benchmark datasets for ML, Dallago *et al.* (2021) recently published the fitness landscape inference for proteins (FLIP) repository, which seeks to address this issue. We make use of two labeled regression datasets from FLIP as well as a third classification dataset from a study related to ML-guided antibody engineering (Mason *et al.*, 2021) (Table 1).

**Table 1.** Description of datasets used in this study

Dataset	Train/test ED split	Residues modified	Training sequences	Validation sequences	Test sequences
GB1 (Wu *et al.*, 2016)	3	4	2691	299	5743
AAV (Bryant *et al.*, 2021)	7	27–42^a^	62 631	7001	12 581
Trastuzumab (Mason *et al.*, 2021)	7	9	11 172	2795	3858

*Note*: Dataset corresponds to wild-type (WT) protein that served as a basis for mutagenesis library generation. Edit distance (ED) threshold splitting for training/validation and test sets are included.

a Length of AAV mutagenized region is variable, as this library contains both insertions and deletions.

#### 2.1.1 GB1 dataset

In the original study (Wu *et al.*, 2016), four positions of GB1, the IgG-Fc binding domain of protein G, were subjected to saturation combinatorial mutagenesis, thus resulting in an overall library diversity of 20^4^ = 160 000 variants. mRNA display, deep sequencing and statistical analysis were used to determine the fitness of the variants. The FLIP subtask chosen for this dataset was ‘Three vs Rest’, a regression task that seeks to predict a value for variant fitness by training (and validating) on sequences with amino acid edit distance 1–3 (ED_1–3_) away from wild-type (WT) and testing on sequences ED_4_ from WT. The subtask was chosen as the quantity of training data is large enough to allow investigation of how training sets of varying size impact model performance when supplemented with NTA. The full dataset for this subtask includes 2691 training, 299 validation and 5743 test sequences. Protein sequences are truncated to the variable region only.

#### 2.1.2 AAV dataset

Bryant *et al.* (2021) performed mutagenesis on a 29 amino acid region of the AAV capsid protein. Some variants contain insertions or deletions, resulting in a maximum of 39 mutations from WT. Variant fitness was assessed via viral production assay, deep sequencing and statistical analysis. The FLIP regression subtask chosen for this dataset was ‘Seven vs Rest’ and seeks to predict a value for variant fitness. This subtask uses variants with an amino acid ED_1–7_ from WT as training and validation data. The test dataset includes variants with amino acid ED_8–39_ from WT. The full dataset for this subtask consists of 62 631 training, 7001 validation and 12 581 test sequences. Protein sequences are truncated to the variable region only. This dataset was chosen to complement the GB1 data, as it offers a larger, more complex training set with a higher number of mutated residues of variable length.

#### 2.1.3 Trastuzumab dataset

Mason *et al.* (2021) performed mutagenesis on nine residues of the heavy chain complementarity determining region 3 (CDRH3) of the therapeutic antibody trastuzumab. Variants were screened for binding or non-binding to the HER2 antigen via mammalian display, fluorescence-activated cell sorting and deep sequencing. A train/test-splitting strategy was developed specifically for this study. To correspond with the other datasets and to maximize the data used, amino acid ED_7_ from WT was chosen as the cutoff between train/validation and test sets. Using edit distance to split training and testing also resembles real-world workflows in which models are trained with a limited number of mutations and used to extrapolate to a larger sequence space. The resulting training set was then balanced by downsampling the number of negatives (non-binders) to match the number of positives (antigen binders) to create a balanced starting point for the synthetic introduction of class imbalance. Twenty percent of the sequences were allocated as a validation set, resulting in a training set of 11 172 sequences, a validation set of 2795 sequences and a test set containing a balanced 1929 positive and 1929 negative sequences. Protein sequences were truncated to the variable region only.

#### 2.1.4 Validation and test sets

NTA trains models on nucleotide sequences and therefore requires the reverse translation of validation and test sets to assess performance. Codon sampling during reverse translation may introduce bias; i.e. certain codons could be more or less represented in the test set compared to the training set (e.g. via random codon sampling). To account for this variability and in accordance with common practice (Krizhevsky *et al.*, 2012), we produce five test set versions, resulting in datasets with the same protein sequence identities, but differing in nucleotide codon distribution. Performance metrics are computed across all five sets, including mean values and standard deviation.

### 2.2 NTA algorithms

NTA implementation raises a number of questions including how codons should be selected, to what extent augmented sequences should differ from one another, and whether the natural AA-to-codon relationship can benefit compared to an arbitrary relationship. In our attempts to answer these questions, we take inspiration from NLP, as codon substitution is analogous to synonym replacement for data augmentation in NLP (Wei and Zou, 2019; Zhang *et al.*, 2015). We explore assembling nucleotide sequences iteratively and randomly as well as using an amino-acid-to-codon relationship with little resemblance to nature and one with a synthetically balanced set of codons. We also test offline (Algorithm 1) and online (Algorithm 2) data augmentation. Offline augmentation increases the overall training set size by copying and transforming each training example multiple times before saving the augmented dataset to disk, prior to model training. Online augmentation, in contrast, does not increase the actual dataset size but transforms mini-batches prior to each gradient descent step during training. Online augmentation may therefore require less disk space but lead to slower training whereas offline may train quicker but require more disk space (Shorten and Khoshgoftaar, 2019).

#### 2.2.1 Offline iterative NTA

As a first proof of concept, we develop a simple offline iterative NTA algorithm that deterministically reverse translates sequence from amino acids to nucleotides by consistently sampling the same codon for a given amino acid (Algorithm 1, Steps 8–11). During augmentation, the reverse translated sequence is copied and a single codon is substituted for one of its analogs. This process continues until all codons available for a given position are exhausted, at which point the algorithm modifies the adjacent codon. Substitutions are ordered according to the cartesian product of position-codon combinations, and this process is repeated until the desired number of augmentations (*n*_aug_) per amino acid sequence is met. The iterative, ordered nature of this method results in modifications concentrated in one region of a sequence.


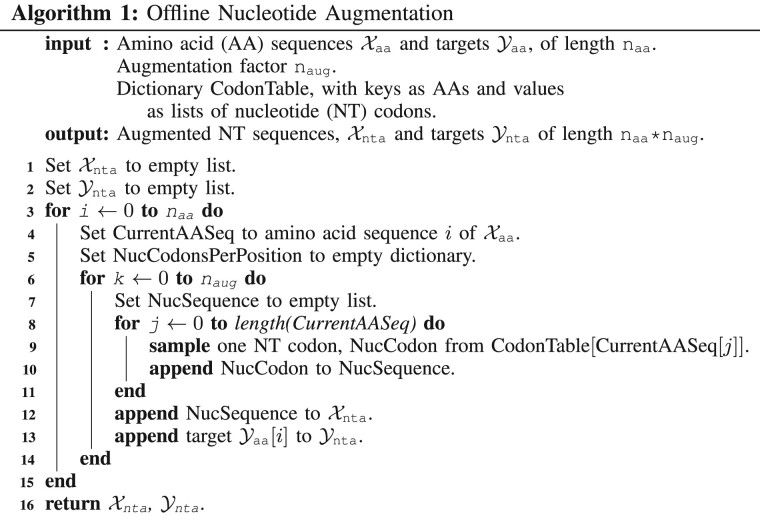


#### 2.2.2 Offline random NTA

To contrast focusing substitutions in a single region, we explore random substitution throughout a sequence. We reverse translate amino acid sequences by randomly selecting corresponding codons for each amino acid (Algorithm 1, Steps 8–11). We repeat this process *n*_aug_ times during augmentation, producing *n*_aug_ synonymous NT sequences per unique amino acid sequence, which results in *n*_aug_, largely different nucleotide sequences.

#### 2.2.3 Online probabilistic NTA

The online NTA implementation datasets for training, validation and testing are reverse translated from amino acids to nucleotides with the random codon sampling approach. During training, a modification probability, *p*_aug_ (between 0 and 1) is randomly generated for each training sequence. In cases where *p*_aug_ exceeds the threshold hyperparameter *t*_aug_ (we use 0.5 for our experiments), the nucleotide sequence is modified according to Algorithm 2 Steps 8–15. This process is repeated for each sequence in a mini-batch prior to taking a gradient descent step during training.

#### 2.2.4 Alternative aminio-acid-to-codon relationships

We also seek to probe how the amino-acid-to-codon relationship impacts learning. The uneven degeneracy in the natural amino acid codon table (e.g. Serine has six codons, where Methionine has one) may bias learning, therefore, we develop a Codon Balance method in which we arbitrarily allocate three codons to each amino acid to create a balanced relationship. Additionally, to determine if using the natural codon relationship provides a benefit over one that is arbitrary, our Codon Shuffle approach shuffles the amino-acid-to-codon relationship while maintaining the number of codons per amino acid. The natural, Codon Balance and Codon Shuffle amino-acid-to-codon relationships can be found in Supplementary Table S1. Offline implementations of Codon Balance and Codon Shuffle make use of iterative sampling and Algorithm 1 while online implementations use random sampling and follow Algorithm 2.


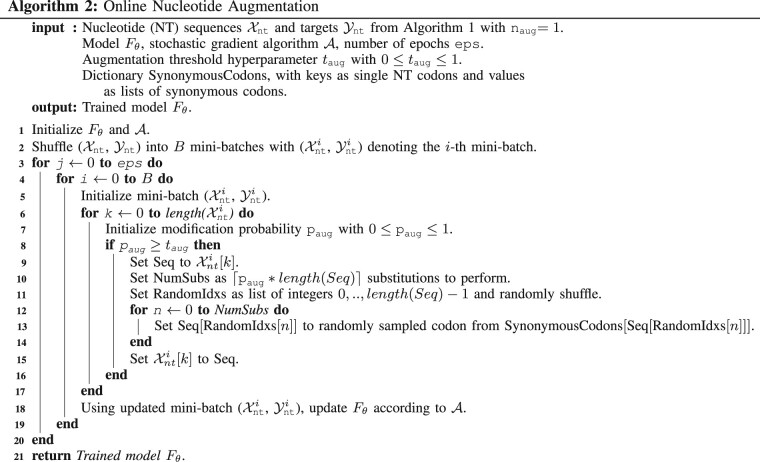


### 2.3 ML models

A transformer (Vaswani *et al.*, 2017) and a convolutional neural network (CNN) were chosen as representative model types for this study due to their widespread use in ML on biological sequence data (Dallago *et al.*, 2021; Mason *et al.*, 2021; Rao *et al.*, 2021, 2019). As the purpose of this work is not to develop the best model for the tasks, but to illustrate the potential of NTA, minimal hyperparameter optimization was performed. It is therefore reasonable to expect additional performance improvement with optimized parameters. The models were written in python using the PyTorch (Paszke *et al.*, 2019) framework.

#### 2.3.1 Transformer model

Two modified transformer (Vaswani *et al.*, 2017) models were created: a larger Transformer-R for the regression tasks and the smaller Transformer-C for classification. In both models, protein and nucleotide sequences are broken into ngrams, categorically encoded and fed to an embedding layer of dimension 256 or 32 followed by positional encoding injection (Vaswani *et al.*, 2017), and then followed by transformer encoder layers with 8 or 2 attention heads with a hidden dimension of 1024 or 128 for Transformer-R and Transformer-C, respectively. The encoder output is flattened then fed to a linear layer with output dimension 1024 or 512 for Transformer-R and Transformer-C, respectively, followed finally by a linear layer with output dimension 1 to predict variant fitness or class. Rectified linear unit (ReLU) is applied as the activation function and dropout (Srivastava *et al.*, 2014) of 0.3 is used throughout the network. To account for variable sequence length resulting from insertions and deletions in the AAV data, sequences are padded to the maximum length and a padding mask is used to prevent the attention mechanism from attending to padded tokens.

#### 2.3.2 CNN model

The CNN architecture is adapted and modified from what was used in the FLIP repository model (Dallago *et al.*, 2021). A two-layer, 1D CNN is used for the regression tasks (CNN-R) and a single layer, 1D CNN for classification (CNN-C). In both CNNs, protein and nucleotide sequences broken into ngrams are one-hot encoded as input. The CNN-R kernel widths are 5 and 3 and the number of convolutional filters 1024 and 512 for the first and second layers. CNN-C uses a kernel width of 5 and 64 filters. All convolutional layers are followed by batch normalization and max pooling. In both networks, the output of the final max pooling layer is mapped to a linear layer with 512 nodes and dropout (Srivastava *et al.*, 2014) 0.3, and final mapping to a linear layer with output dimension 1. ReLU is applied as the activation function throughout. Sequence padding and mask are applied to the AAV dataset to handle sequences of variable length.

#### 2.3.3 ngram sequence encoding

NTA requires reverse translation of amino acids to nucleotides, which increases the sequence length 3-fold. To probe how input format affects performance, three nucleotide ngram encoding schemes were tested: unigrams, trigrams, and tri+unigrams, which concatenates trigrams and unigrams. Each encoding scheme has a different vocabulary or set of unique ‘words’ (ngrams). The unigram vocabulary has four elements: A,T,G,C. The trigram vocabulary consists of the 64 possible nucleotide codons. The tri+unigram vocabulary combines the two for a total of 68 elements. Protein sequences were encoded as unigrams with a vocabulary of 21, one character for each of the canonical amino acids plus the UNK token. It is worth noting that the nucleotide trigram and amino acid unigram encodings share the same sequence length, albeit a different vocabulary size. After breaking a sequence into ngrams, categorical encoding and one-hot encoding are applied for the Transformer and CNN, respectively.

#### 2.3.4 Baselines, performance metrics and model training

We compare NTA to two baselines: the amino acid (AA) baseline, in which models are trained with the original amino acid datasets and the DNA baseline, in which models are trained on the equivalent DNA sequences without augmentation. Consistent with common practice, validation and test sets were reverse translated without augmentation using the codon sampling method (iterative or random) mirroring that of the experimental setup.

The loss function used for the regression tasks is mean squared error and in accordance with previous work with the FLIP repository (Dallago *et al.*, 2021), Spearman’s Rho is used to assess model performance. Binary cross entropy is used for the classification task and the Matthews correlation coefficient (MCC), which ranges from −1 to 1, is selected as the performance metric as it is an appropriate summary metric for binary classification of imbalanced datasets (Chicco and Jurman, 2020). Stochastic gradient descent with momentum of 0.9 is used as the optimizer. Models were trained using the ETH Zurich Euler Cluster with 1 GPU (Nvidia GTX 1080, GTX 1080 Ti or V100).

For offline augmentation, five different *n*_aug_ values were evaluated on the GB1 (2, 5, 10, 50 and 100), four on the AAV (2, 5, 10 and 25) data and three values (2, 5 and 10) for the Trastuzumab data. Baselines and augmented sets were trained with early stopping based on validation performance to replicate typical learning scenarios in which a test set is unavailable. We then used online NTA to explore whether performance gains result from the augmentation transformation (codon substitution) or from the simple upsampling of training data. As online augmentation transforms sequences without increasing dataset size, we forgo early stopping and train the same number of epochs for augmented and baseline cases to ensure models are exposed to the same number of data points.

## 3 Results

We begin by describing the experimental setup for the regression tasks and presenting results on the GB1 dataset with the CNN-R model for each of the developed NTA methods (Fig. 1). Transformer-R GB1 results are included in the Supplementary information (Supplementary Fig. S1) and largely mirror CNN-R. We then present NTA results on the AAV and Trastuzumab datasets.

### 3.1 Regression variant fitness prediction data subsetting

To probe how data quantity impacts ML models, it is common to truncate a training set to different sizes and assess model performance. This type of analysis is useful since biological data collection is time- and resource-intensive, and thus can aid in experimental design.

To test how NTA performs with differing amounts of initial sequence diversity, training datasets were truncated into separate subsets consisting of 1%, 5%, 10%, 25%, 50%, 75% and 100% of the available training data. A 0.5% fraction of the AAV dataset was also created due to the dataset’s larger total size. The truncated sets correspond to a range of either 26–2691 (GB1) or 313–62 631 (AAV) sequences. In an attempt to mirror the distribution found in the full training and validation sets (as defined by the FLIP repository), sequences were binned by fitness value, a continuous value approximating mutant fitness experimentally determined in each study. Truncation was performed using train_test_split with ‘stratify’ option from sklearn (version 0.23.2) to maintain the ratio of binned sequences during truncation.

### 3.2 GB1 fitness prediction with NTA

**Fig. 2.**
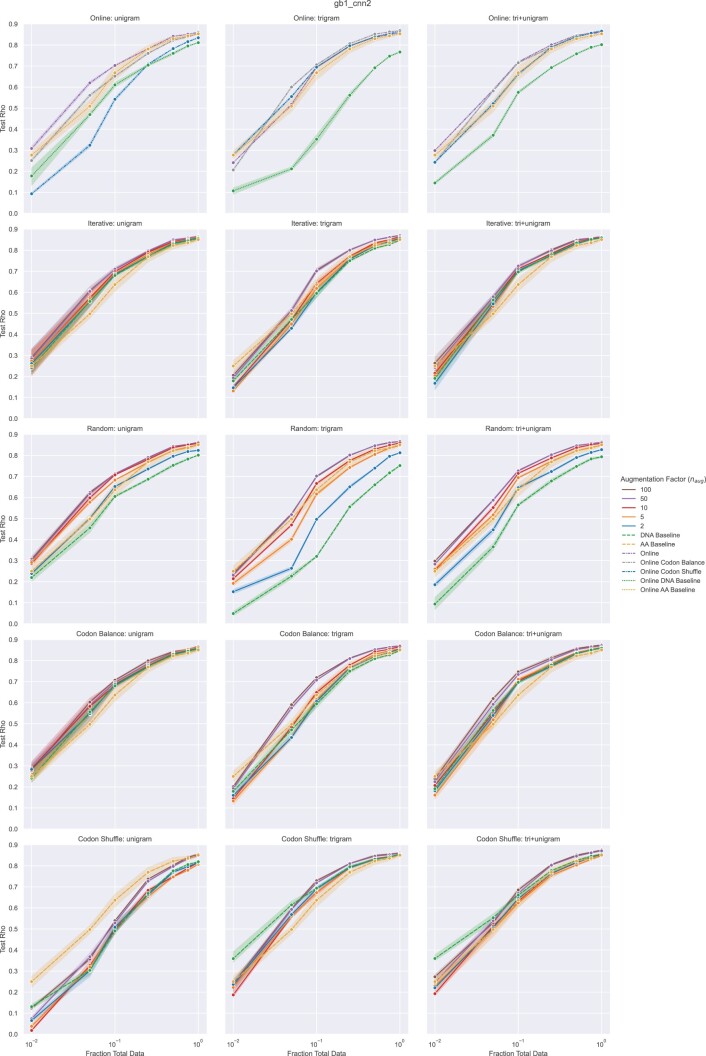
CNN-R fitness prediction performance (Spearman’s Rho) of GB1 mutants as a function of the number of training samples used for model training. Points correspond to mean performance and shaded regions to 95% confidence interval across three random seeds. Baselines include models trained on amino acid sequences (yellow) and models trained on DNA sequences without augmentation (green). Rows correspond to NTA implementations tested. Columns correspond to encoding schemes. Offline NTA performance is reported for a range of augmentation factors (*n*_aug_). Online NTA performance is reported for the various amino-acid-to-codon relationships

In general, NTA improves GB1 prediction over baselines for almost every implementation and encoding scheme, and notably for models trained with small datasets (Fig. 2, Supplementary Fig. S1). For example, the trigram CNN-R with 25% training data (672 sequences) results in a test Rho of 0.75 ± 0.01 on the DNA baseline, 0.74 ± 0.00 on the AA baseline and 0.80 ± 0.00 with iterative NTA and an *n*_aug_ of 50. NTA continues to result in improvements for most scenarios using larger training sets as well. The best-performing model described in Dallago *et al.* (2021) (CNN) achieves 0.83 on the full FLIP dataset. Remarkably, using only 50% training data and iterative NTA, our CNN-R achieves 0.85 ± 0.00 for each ngram encoding at an *n*_aug_ of 100. Notable differences between NTA implementations are discussed in the following sections.

#### 3.2.1 Iterative versus random sampling in offline augmentation

The largest differences occur for the non-augmented DNA baselines, with the random method baseline significantly below and the iterative method baseline closely matching the amino acid baseline. This may be due to the fact that the iterative method consistently uses the same codon for a given amino acid during reverse translation and only selects an alternative codon during augmentation, ensuring a given amino acid is represented similarly across train, validation and test sets. Random sampling, in contrast, randomly selects different codons from available codon space each time an amino acid is reverse translated. For two protein sequences differing by one amino acid, for example, reverse translation via random sampling may produce two nucleotide sequences with few codons in common. Therefore, codons can be over/under represented in the training set compared to the validation/test sets, potentially resulting in poor predictions. One would expect this phenomena to decrease in magnitude as dataset size increases and codons are more evenly sampled. Indeed when random augmentation is applied to the GB1 data, performance gains quickly match or surpass those seen with iterative augmentation.

#### 3.2.2 Alternative amino-acid-to-codon relationships

The Codon Balance and Codon Shuffle methods are used to investigate the impact of the amino-acid-to-codon relationship. In general, offline implementations that sample iteratively from codon space (iterative, Codon Balance and Codon Shuffle) and online implementations, which sample probabilistically from codon space are comparable. Codon Balance (Fig. 2, 4th row from top) outperforms the natural amino acid to nucleotide relationship for trigram and tri+unigram encoding schemes in both on- and offline implementations; however, the natural codon degeneracy outperforms Codon Balance for unigram encoding. In contrast, Codon Shuffle (Fig. 2, bottom row) performs poorly most acutely for unigram encoding followed by tri+unigram encoding. This makes intuitive sense as natural degeneracy encodes amino acids by codons oftentimes differing by only a single base; a connection that is lost as codons are arbitrarily shuffled. When each codon is encoded as a single trigram, the performance drop is much less acute.

#### 3.2.3 Online augmentation

To test the hypothesis that improvements result from the codon substitution transformation instead of a simple upsampling of training data, we develop an online augmentation protocol that trains models under baseline and augmented conditions with equal training set sizes for the same number of epochs. In testing natural, Codon Balance and Codon Shuffle amino-acid-to-codon relationships, online NTA outperforms baselines in almost every case across encodings and training set subsampling regimes (Fig. 2, top row), which supports our hypothesis. Additionally, similar behavior is observed between offline and online augmentation when using alternate codon relationships. Finally, similar to random offline augmentation, the online DNA baseline performs much poorer than the amino acid baseline likely resulting from the random/probabilistic sampling approaches.

### 3.3 AAV fitness prediction with NTA

**Fig. 3.**
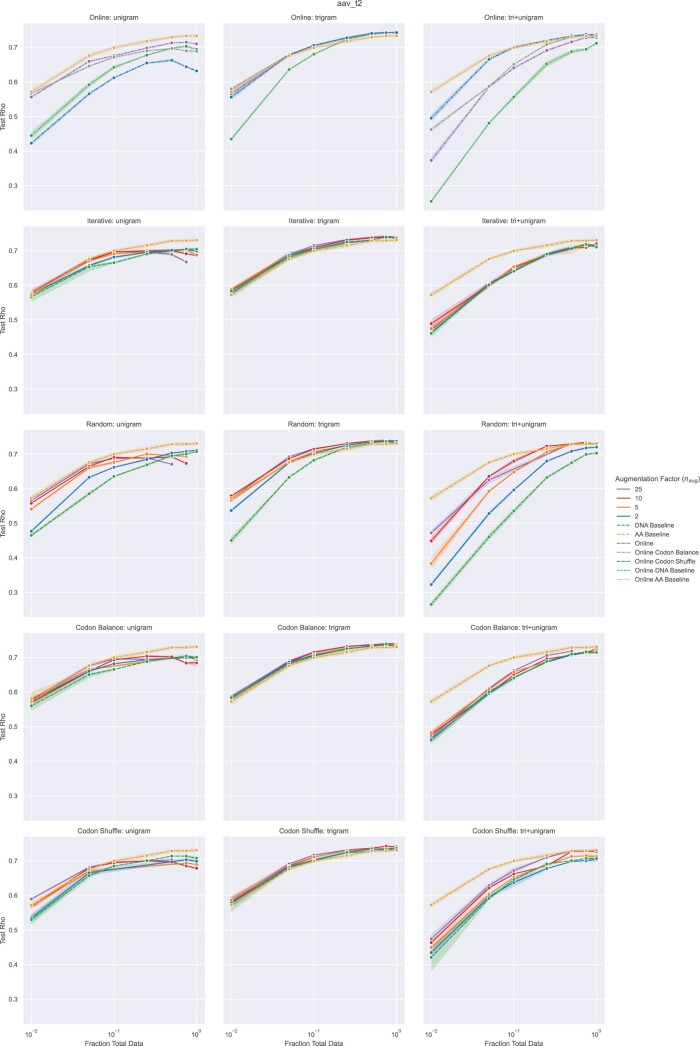
Transformer-R fitness prediction performance (Spearman’s Rho) of AAV mutants as a function of the number of training samples used for model training. Points correspond to mean performance and shaded regions to 95% confidence interval across three random seeds. Baselines include models trained on amino acid sequences (yellow) and models trained on DNA sequences without augmentation (green). Rows correspond to NTA implementations tested. Columns correspond to encoding schemes. Offline NTA performance is reported for a range of augmentation factors (*n*_aug_). Online NTA performance is reported for the various amino-acid-to-codon relationships

In contrast to GB1, NTA performance gains on the AAV task are dependent on the selected encoding scheme (Fig. 3, Supplementary Fig. S2). Improvements over baseline for the Transformer-R are observed with trigram encoding, which maintains the same input shape as amino acid sequences and NTA performs worse than baseline for unigram and tri+unigram encoding. This discrepancy is most likely attributable to the 3-fold (unigram) or more (tri+unigram) increase in input length, which resulted in training instability and required gradient clipping. Additionally, the longer input length requires a smaller batch size, increasing training time and a maximum training time of 48 h was set for larger *n*_augs_. For online augmentation, we found that substituting more than a quarter of the codons in a given sequence was detrimental to performance in certain cases and therefore fixed the hyperparameter NumSubs (Algorithm 2) at 0.25 for the AAV dataset. These results highlight the importance of encoding and architecture selection for a given learning task. Differences observed between NTA implementations resemble those discussed for GB1.

### 3.4 Class-imbalanced binding classification with NTA

Protein engineering through the screening of mutagenesis libraries often results in a substantial fraction of low- or non-functional variants and a comparatively low fraction of variants with enhanced properties, yielding imbalanced datasets for ML (Wittmann *et al.*, 2021b). To test if NTA can improve learning on data with heavy class imbalance, we first create a balanced training set (Section 2.1.3) then artificially imbalance the data by down-sampling the number of positive examples (antibody variants binding to target antigen) to range from 10% to 100% of the number of negative sequences (antibody variants non-binding to antigen). Similar to our regression experiments, following downsampling, validation sets were split using sklearn’s ‘train_test_split’ function with ‘stratify’ option to produce validation sets 20% the size of each training set while maintaining the respective class imbalance. Baselines were collected on the imbalanced data for both amino acid and non-augmented DNA sequences. NTA was applied only to the minority class (antigen-binding sequences) of the training set in accordance with common practice (Afzal *et al.*, 2019; Saini and Susan, 2019; Shamsolmoali *et al.*, 2021). The minority class was augmented by a factor of 2, 5 and 10. Further augmentation beyond 10 generally caused a drop in performance. The majority class (antigen non-binding) sequences were reverse translated without augmentation. PyTorch’s WeightedRandomSampler was used to class balance mini-batches as much as possible during training as this was found to result in better performance.

#### 3.4.1 Imbalanced classification results

**Fig. 4.**
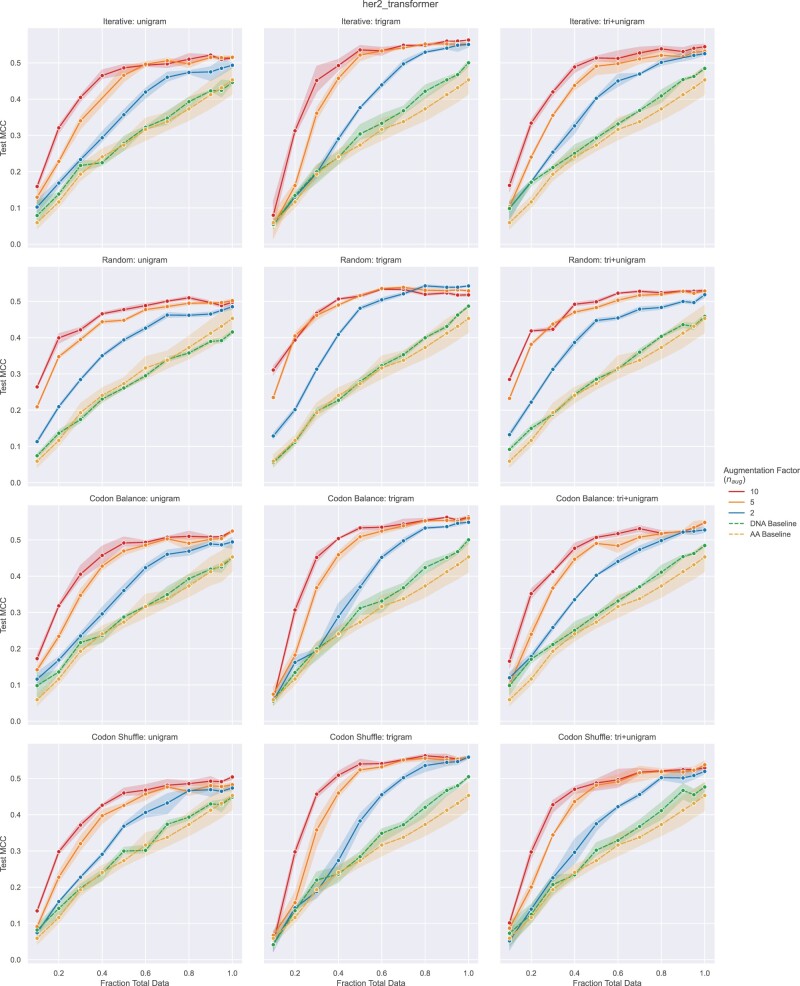
Transformer-C binary classification (antigen binding and non-binding) prediction performance (Matthews correlation coefficient) of antibody variants as a function of the ratio of positive (minority class) to negative (majority class) sequences in the training set. Points correspond to mean performance and shaded regions to 95% confidence interval across three random seeds. Baselines include models trained on amino acid sequences (yellow) and models trained on DNA sequences without augmentation (green). Rows correspond to NTA implementations tested. Columns correspond to encoding schemes. Offline NTA performance is reported for a range of augmentation factors (*n*_aug_)

NTA yields significant improvements on imbalanced antibody–antigen binding data for every model-encoding scheme tested with dramatic improvements under heavy class imbalance. For example with a 0.3 positive to negative ratio, iterative NTA improves MCC from 0.17 ± 0.03 and 0.19 ± 0.02 (AA and DNA baselines, respectively) to 0.44 ± 0.02 at an augmentation factor of 10 with the trigram Transformer-C (Fig. 4). Performance generally increases with an augmentation factor up to 10, at which point overfitting starts to be observable in CNN-C (Supplementary Fig. S3) and continues as augmentation was pushed further. The trigram Transformer-C was found to perform the best and the unigram CNN-C the poorest. Overall, these results demonstrate the ability of NTA to aid in learning from class-imbalanced data. Online augmentation was not tested for Trastuzumab data as online augmentation would not remediate class imbalance without prior training set balancing, confounding the comparison.

## 4 Discussion

The collection of protein genotype–phenotype data is time- and resource-intensive, thus representing a critical bottleneck for ML-guided protein engineering. Previous work has sought to counter data limitations with GANs (Han *et al.*, 2019; Li and Zhang, 2022) and language models (Lu *et al.*, 2020; Shen *et al.*, 2021), however, GANs are limited to the domain in which they are trained and language models may not always be appropriate for protein engineering workflows introducing large libraries of non-natural mutations into a small number of residues of a protein (Biswas *et al.*, 2021; Dallago *et al.*, 2021; Wittmann *et al.*, 2021a). For example, Dallago *et al.* (2021) find that for synthetic mutagenesis datasets focusing on a single protein, language models can be outperformed by smaller, more focused models. To date, the field lacks the simple, easy-to-use data augmentation techniques that are commonly found in the fields of computer vision and NLP. Towards this end, we develop here offline and online NTA, which leverages the nucleotide codon degeneracy of protein sequences to augment datasets for ML. We apply NTA to three labeled datasets for ML-guided protein engineering, two of which (GB1 and AAV) have been established as benchmarks by the recent FLIP repository (Dallago *et al.*, 2021).

We find that NTA yields large gains when limited training data is available (e.g. 10–10^3^ sequences), while still enhancing performance as more data is collected. This is particularly useful for protein engineering workflows unable to generate high-throughput data (i.e. variant stability and affinity characterization or cell-based assays). We also find that NTA serves as a promising method to improve learning on class-imbalanced data, which is a common occurrence in protein engineering experiments (Wittmann *et al.*, 2021b). Our findings are generally consistent between the Transformer and CNN models and small differences are observed between amino acids and each DNA ngram encoding when the input sequence length is short. For longer sequences, however, significant differences are observed between encoding schemes, highlighted by the AAV results. The 3-fold (unigram) or longer (tri+unigram) sequence length coupled with large regions of insertions and deletions, requiring sequence padding, can complicate learning, which highlights the importance of encoding scheme and model architecture selection.

We further characterize our codon substitution-based augmentation approach with iterative and random sampling, alternative amino-acid-to-codon relationships, and offline and online implementations. Iteratively sampling from codon space is comparable to training on amino acids, especially with trigram encoding (thereby preserving the same input shape), while random sampling hinders performance without augmentation, however, both sampling approaches yield comparable results when augmentation is executed. Codon Balance and Codon Shuffle demonstrate the importance of the amino-acid-to-codon relationship in NTA. For example, training with DNA unigrams and Codon Shuffle yields poor performance while Codon Balance largely matches or exceeds other approaches across encodings. Our results appear to suggest one could potentially optimize the amino-acid-to-codon relationship (manually or computationally) to best serve the learning task at hand. Finally, our online augmentation setup supports the hypothesis that the codon substitution transformation, as opposed to simply upsampling data, contributes to performance gains.

The development and testing of NTA naturally led to the characterization of a variety of design choices. As a result of our experiments, for those seeking to apply NTA, we recommend testing a variety of encoding schemes, implementations and hyperparameters (i.e. *n*_aug_, *t*_aug_ and *p*_aug_).

NTA can be easily applied to most protein-ML workflows and requires minimal additional resources. One potential downside of NTA is that it prohibits the use of models pretrained with amino acids. It is worth noting that NTA could be applied to additional areas of ML-guided protein engineering, such as predictions of stability, immunogenicity and subcellular localization (Li *et al.*, 2021; Rao *et al.*, 2019). Finally, NTA could supplement contrastive learning and be combined with or used to aid in the training of protein language models (Lu *et al.*, 2020; Shen *et al.*, 2021). Future work may also seek to improve the NTA algorithm and its application for specific use cases.

## Supplementary Material

vbac094_Supplementary_Data
