# Supplementary material for: Nucleotide augmentation for machine learning-guided protein engineering
Source: Bioinform Adv. 2022 Dec 9;3(1):vbac094. doi: 10.1093/bioadv/vbac094 (PMC9843584; doi:10.1093/bioadv/vbac094)
Supplement: vbac094_Supplementary_Data [file vbac094_supplementary_data.zip › nta_supplement.pdf]

Supplemental Information for Nucleotide augmentation for machine learning-guided protein engineering

Authors: Mason Minot, Sai T. Reddy*

Affiliation:
ETH Zurich, Department of Biosystems Science and Engineering, Basel, 4058, Switzerland

Correspondence: sai.reddy@ethz.ch

Table ST1. Amino-Acid-to-Codon Relationships

Natural Codon Table						
A	GCT	GCC	GCA	GCG		
R	CGT	CGC	CGA	CGG	AGA	AGG
N	AAT	AAC				
D	GAT	GAC				
C	TGT	TGC				
Q	CAA	CAG				
E	GAA	GAG				
G	GGT	GGC	GGA	GGG		
H	CAT	CAC				
I	ATT	ATC	ATA			
L	CTT	CTC	CTA	CTG	TTA	TTG
K	AAA	AAG				
M	ATG					
F	TTT	TTC				
P	CCT	CCC	CCA	CCG		
S	TCT	TCC	TCA	TCG	AGT	AGC
T	ACT	ACC	ACA	ACG		
W	TGG					

Y	TAT	TAC				
V	GTT	GTC	GTA	GTG		
Codon Balance Table						
A	GCT	GCC	GCA			
R	CGT	CGC	CGA			
N	AAT	AAC	GCG			
D	GAT	GAC	CGG			
C	TGT	TGC	AGA			
Q	CAA	CAG	AGG			
E	GAA	GAG	GGG			
G	GGT	GGC	GGA			
H	CAT	CAC	CTG			
I	ATT	ATC	ATA			
L	CTT	CTC	CTA			
K	AAA	AAG	TTA			
M	ATG	TTG	CCG			
F	TTT	TTC	TCG			
P	CCT	CCC	CCA			
S	TCT	TCC	TCA			
T	ACT	ACC	ACA			
W	TGG	AGT	AGC			
Y	TAT	TAC	ACG			
V	GTT	GTC	GTA			
Codon Shuffle Table						
A	ATC	TCA	GAG	GAA		
R	GTA	TTG	TTA	CAG	AGG	ACG
N	TGC	AGC				
D	AAG	GCC				
C	ATG	CAT				
Q	TCG	ATA				

E	GGC	GCT				
G	AAC	CAC	GTT	TCT		
H	CTA	CGA				
I	TCC	GTG	ACA			
L	GAT	CTC	CGG	GAC	CCG	CTG
K	AAA	CGT				
M	GGT					
F	CAA	CCC				
P	TAT	TGG	TGT	CCA		
S	TAA	GTC	GCG	AAT	GCA	TAC
T	TTC	ACT	AGT	TAG		
W	AGA					
Y	CTT	GGA				
V	CGC	ACC	TGA	TTT		

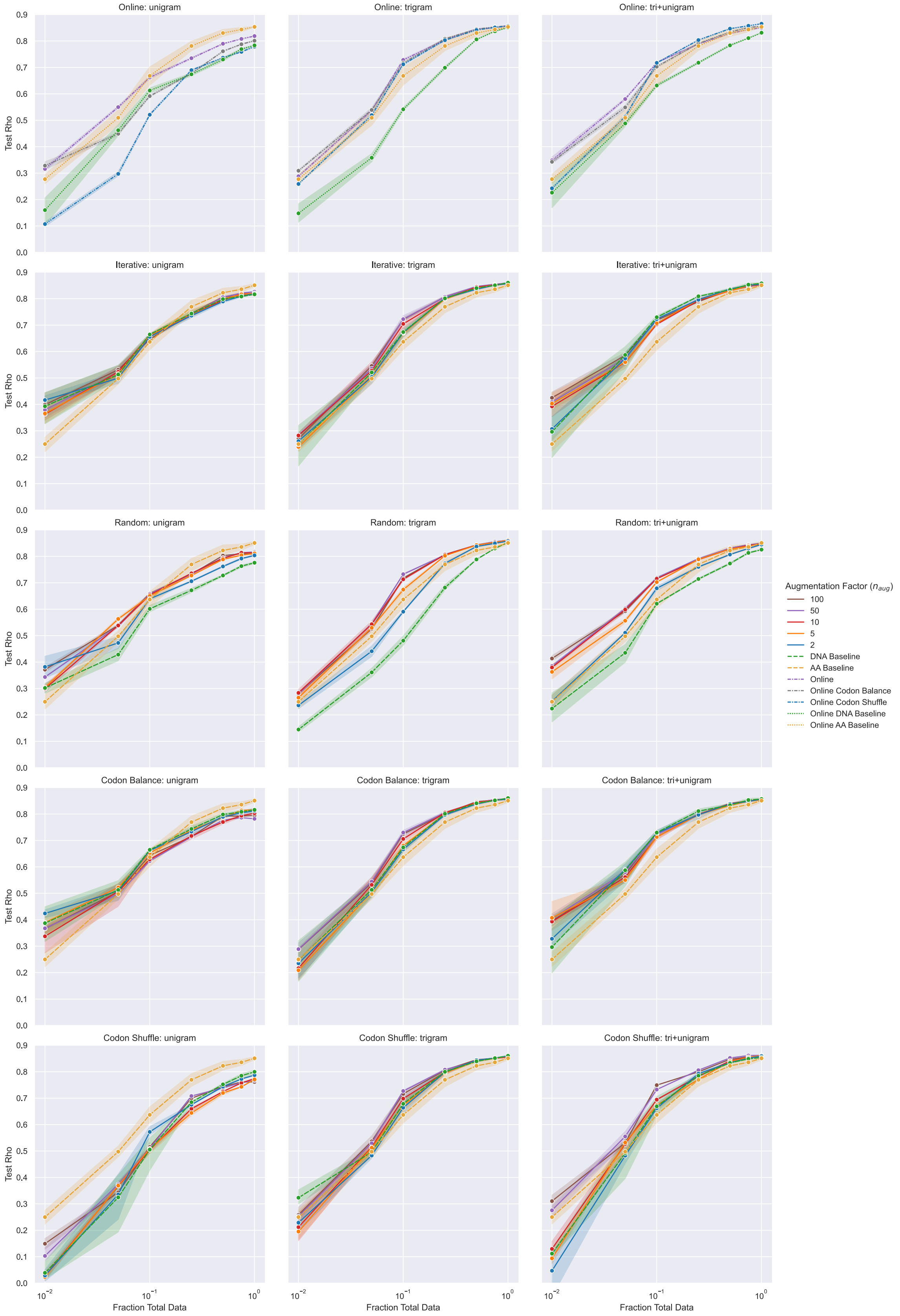

Fig. S1. Transformer-R fitness prediction performance (Spearman's Rho) of GB1 mutants as a function of the number of training samples used for model training. Points correspond to mean performance and shaded regions to 95 % confidence interval across 3 random seeds. Baselines include models trained on amino acid sequences (yellow) and models trained on DNA sequences without augmentation (green). Rows correspond to NTA implementations tested. Columns correspond to encoding schemes. Offline NTA performance is reported for a range of augmentation factors (n_{aug}). Online NTA performance is reported for the various amino-acid-to-codon relationships.

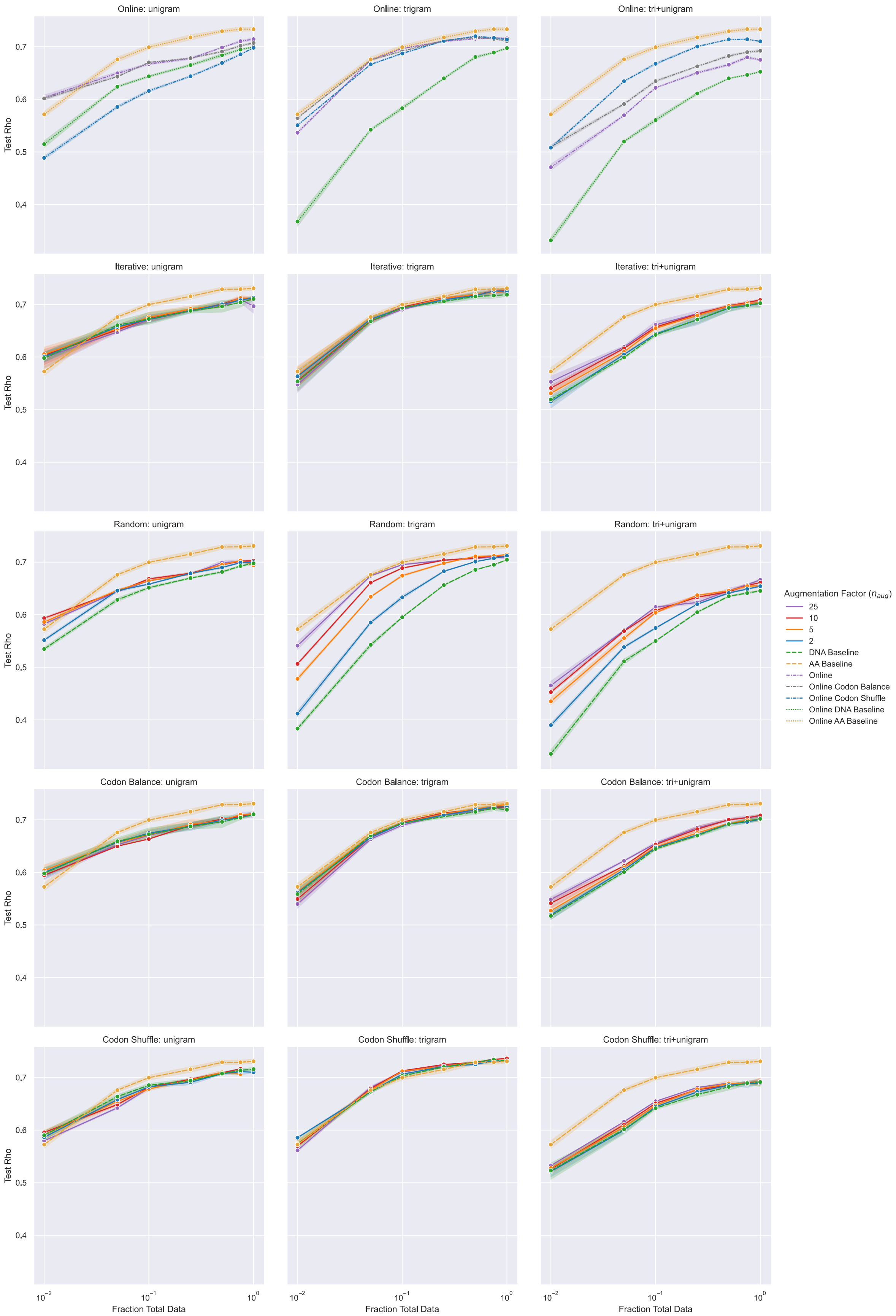

Fig. S2. CNN-R fitness prediction performance (Spearman's Rho) of AAV mutants as a function of the number of training samples used for model training. Points correspond to mean performance and shaded regions to 95 % confidence interval across 3 random seeds. Baselines include models trained on amino acid sequences (yellow) and models trained on DNA sequences without augmentation (green). Rows correspond to NTA implementations tested. Columns correspond to encoding schemes. Offline NTA performance is reported for a range of augmentation factors (n_{aug}). Online NTA performance is reported for the various amino-acid-to-codon relationships.

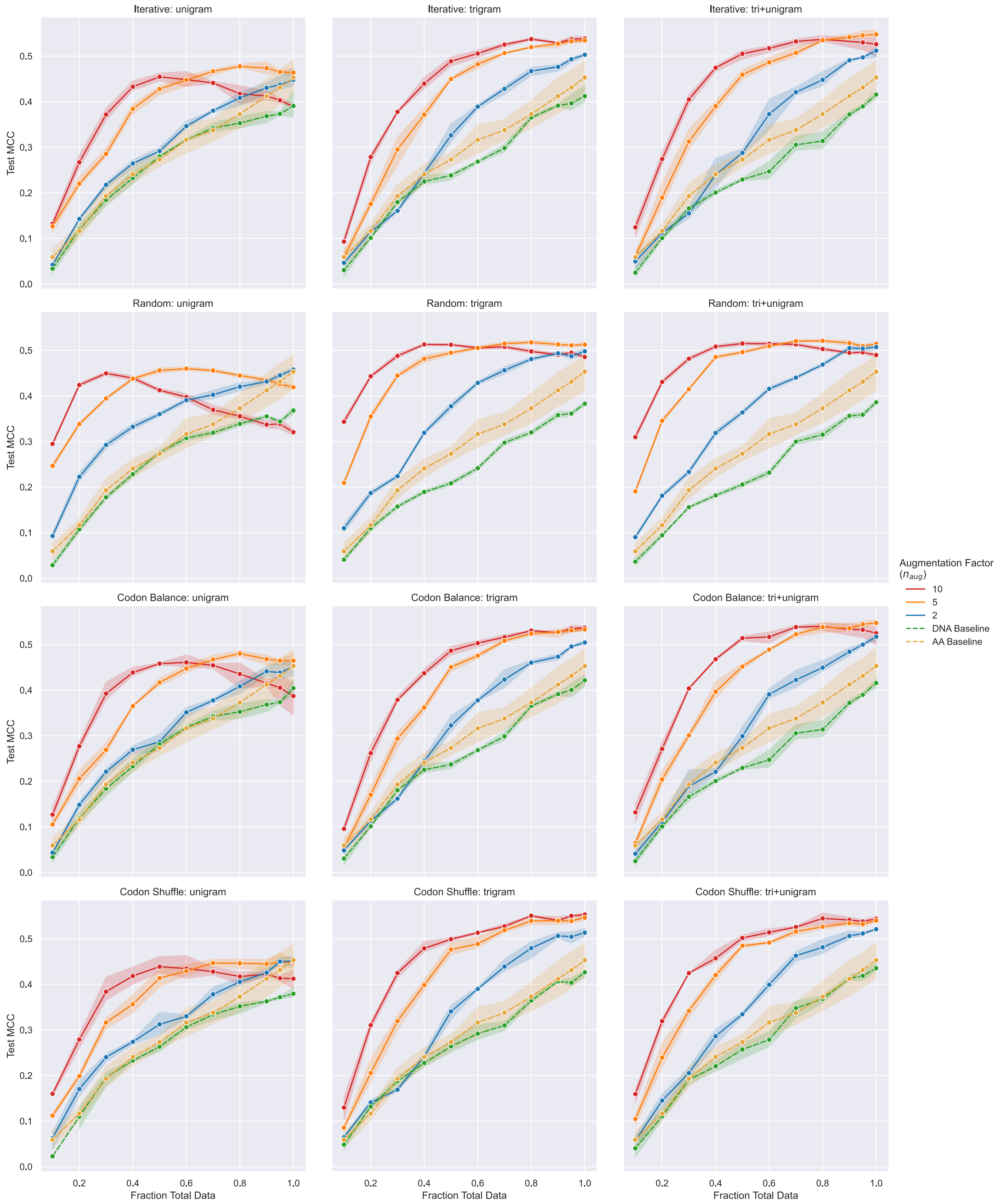

Fig. S3. CNN-C binary classification (antigen binding and non-binding) prediction performance (Matthews Correlation Coefficient) of antibody variants as a function of the ratio of positive (minority class) to negative (majority class) sequences in the training set. Points correspond to mean performance and shaded regions to 95 % confidence interval across 3 random seeds. Baselines include models trained on amino acid sequences (yellow) and models trained on DNA sequences without augmentation (green). Rows correspond to NTA implementations tested. Columns correspond to encoding schemes. Offline NTA performance is reported for a range of augmentation factors (n_{aug}).
