# Supplementary figures and images for: Nucleotide augmentation for machine learning-guided protein engineering
Source: Bioinform Adv. 2022 Dec 9;3(1):vbac094. doi: 10.1093/bioadv/vbac094 (PMC9843584; doi:10.1093/bioadv/vbac094)

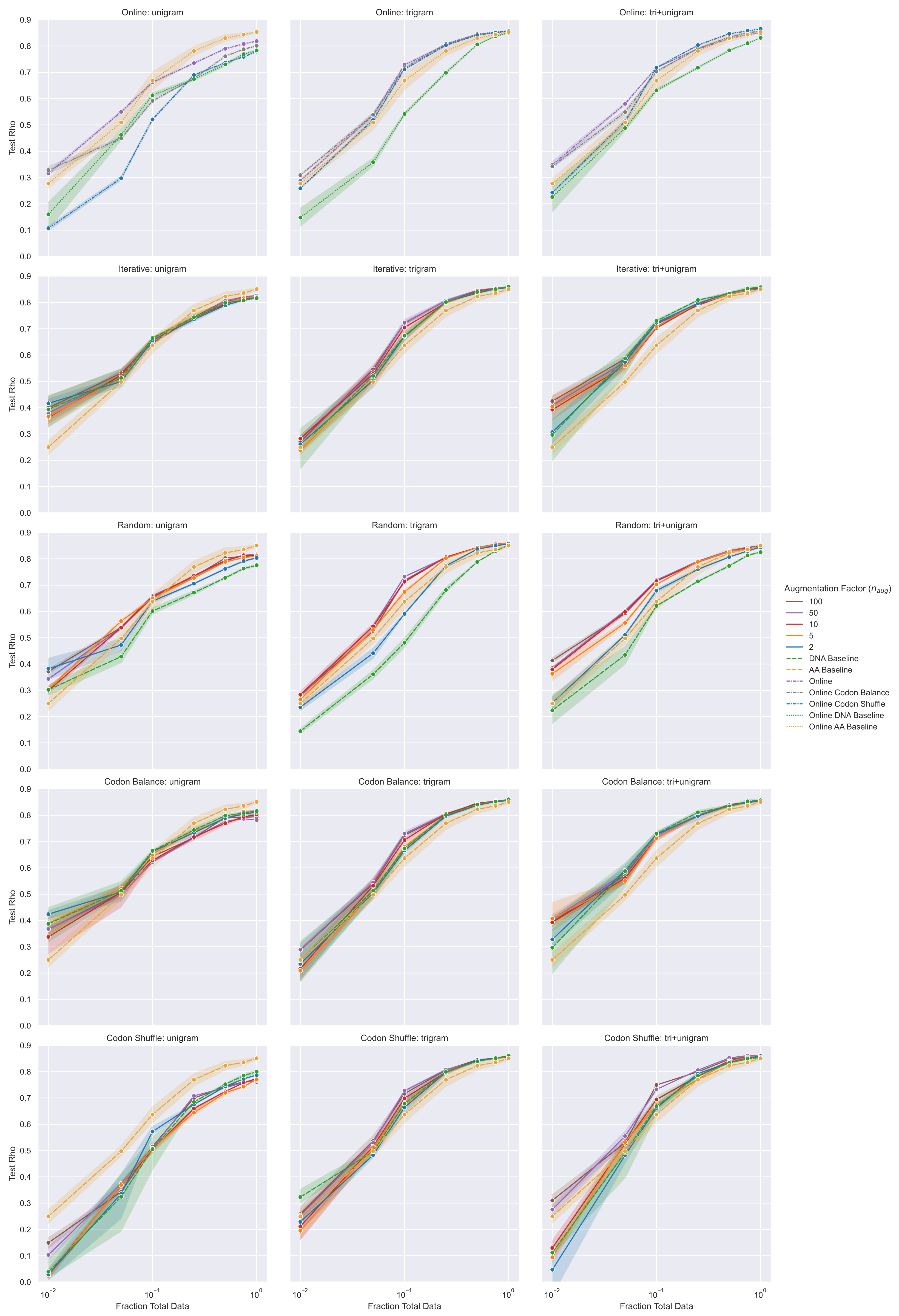

Supplement: vbac094_Supplementary_Data [file vbac094_supplementary_data.zip › FigS1.pdf]
